# Author Correction: Protein interaction network of alternatively spliced isoforms from brain links genetic risk factors for autism

**DOI:** 10.1038/s41467-023-36264-y

**Published:** 2023-02-02

**Authors:** Roser Corominas, Xinping Yang, Guan Ning Lin, Shuli Kang, Yun Shen, Lila Ghamsari, Martin Broly, Maria Rodriguez, Stanley Tam, Shelly A. Wanamaker, Changyu Fan, Song Yi, Murat Tasan, Irma Lemmens, Xingyan Kuang, Nan Zhao, Dheeraj Malhotra, Jacob J. Michaelson, Vladimir Vacic, Michael A. Calderwood, Frederick P. Roth, Jan Tavernier, Steve Horvath, Kourosh Salehi-Ashtiani, Dmitry Korkin, Jonathan Sebat, David E. Hill, Tong Hao, Marc Vidal, Lilia M. Iakoucheva

**Affiliations:** 1 Department of Psychiatry, University of California San Diego, La Jolla, California 92093 USA; 2 Center for Cancer Systems Biology (CCSB) and Department of Cancer Biology, Dana-Farber Cancer Institute, Boston, Massachusetts 02115 USA; 3 Department of Genetics, Harvard Medical School, Boston, Massachusetts 02115 USA; 4 Donnelly Centre and Departments of Molecular Genetics & Computer Science, University of Toronto, and Samuel Lunenfeld Research Institute, Mt. Sinai Hospital, Toronto, Ontario M5S 3E1 Canada; 5 Department of Medical Protein Research, VIB, and Department of Biochemistry, Faculty of Medicine and Health Sciences, Ghent University, Ghent, B-9000 Belgium; 6 Department of Computer Science and Informatics Institute, University of Missouri, Columbia, Missouri 65203 USA; 7 Beyster Center for Genomics of Psychiatric Diseases and Department of Psychiatry, University of California San Diego, La Jolla, California 92093 USA; 8 New York Genome Center, New York, New York, 10013 USA; 9 Department of Human Genetics and Biostatistics, University of California, Los Angeles, California 90095 USA; 10 Present Address: Columbia University, New York, New York, 10032 USA; 11 Present Address: Salk Institute for Biological Studies, La Jolla, California 92037 USA; 12 Present Address: Department of Psychiatry, University of Iowa, Iowa City, Iowa 52242 USA; 13 Present Address: Division of Science and Math, and Center for Genomics and Systems Biology, New York University Abu Dhabi, P.O. Box 129188, Abu Dhabi, United Arab Emirates

Correction to: *Nature Communications* 10.1038/ncomms4650, published online 11 April 2014

Since the publication of this work, Shelly A. Wanamaker has changed their name from Shelly A. Trigg. This has now been amended.

